# Impact of *Terminalia chebula* Retz polysaccharide and *Rubia cordifolia* L. processed *Terminalia chebula* Retz polysaccharides on cyclophosphamide-induced immunosuppression in Chinese yellow quail

**DOI:** 10.3389/fcimb.2025.1620053

**Published:** 2025-07-04

**Authors:** Jing Wang, Tiejin Tong, Qiang Wu, Min He

**Affiliations:** ^1^ School of Advanced Agricultural Sciences, Yibin Vocational and Technical College, Yibin, China; ^2^ College of Veterinary Medicine, Sichuan Agricultural University, Chengdu, China

**Keywords:** *Terminalia chebula* Retz, *Rubia cordifolia* L. processed *Terminalia chebula* Retz polysaccharide, transcriptomics, immunosuppression, Chinese yellow quail

## Abstract

**Introduction:**

Immunosuppression in poultry production, particularly in high-value species like the Chinese yellow quail (Coturnix japonica), increases disease susceptibility and economic losses. Plant-derived polysaccharides, such as those from *Terminalia chebula* Retz (TC) and *Rubia cordifolia* L. processed Terminalia chebula Retz polysaccharide (RTCP), offer promising alternatives due to their ability to enhance host defense mechanisms without adverse effects. This study investigates RTCP’s efficacy in mitigating cyclophosphamide (CTX)-induced immunosuppression in quails, focusing on immune function restoration.

**Methods:**

One hundred and twenty 21-day-old healthy quails were randomly divided into six groups (n=20/group): a blank control (saline), a model group (saline + CTX), and four RTCP-treated groups (0.25, 0.5, 0.75, 1 g/kg + CTX). CTX (80 mg/kg) was administered intramuscularly (days 4–7) to induce immunosuppression, except in the blank control. Spleen indices, histology, cytokine profiles, antibody titers, GATA-3/T-bet mRNA expression, and transcriptional networks were analyzed.

**Results:**

CTX significantly reduced spleen weight, organ indices, cytokine levels, and antibody titers (P<0.05). Compared to the model group, 0.5 g/kg RTCP restored spleen morphology and function, elevated cytokines (e.g., IL-2, IFN-γ) and immunoglobulins, and upregulated complement components (C3, C5) and acute-phase proteins, enhancing T-cell and B-cell activation (P<0.05). RTCP also rebalanced the Th1/Th2 axis by normalizing the T-bet/GATA-3 ratio, indicating immune homeostasis recovery.

**Discussion:**

RTCP effectively counteracted CTX-induced immunosuppression by modulating innate and adaptive immunity, including complement activation and Th1/Th2 balance. These findings highlight RTCP’s potential as a natural immunomodulator in poultry. Further research should optimize dosing and explore gut-microbiota-immune interactions to enhance therapeutic applications.

## Introduction

Currently, in poultry farming, immunosuppression is a major concern and caused by multiple stressors, including pathogen invasion, overuse of antibiotics and chemicals, environmental pollutants, and management-related stress (Karam and Wali, 2015). Poultry with compromised immune defenses generally display increased susceptibility to secondary infections, diminished adaptive immunity, and reduced responsiveness to prophylactic vaccines, which contribute to significant economic losses within the industry (Sharma et al., 2000). The prolonged or excessive use of antiviral drugs and vaccines, which served as common strategies for controlling disease outbreaks, raises concerns regarding residue accumulation and the generation of antimicrobial resistance, leaving additional risks for food safety and public health. The Chinese yellow quail (Coturnix japonica), a commercially valuable breed derived from the Korean Ryuseong lineage through selective mutation, plays a prominent role in Asian markets relying on its rapid maturation, high reproductive capacity, and efficient feed conversion. However, quail production is often impeded by the issue of immunosuppression, which declines disease resistance, growth performance, and overall flock productivity (Nain et al., 2011). Presently, research on immunomodulatory interventions remains mainly focused on murine and chicken models, highlighting a critical gap for species-specific strategies of quail therapy. Therefore, it is imperative to develop immunomodulatory drugs or feed additives that can enhance the innate immunity and lower immunosuppressive stressors in this underexplored avian species.

Polysaccharides are a very promising group of immunostimulants because they can boost the body’s defense systems with few side effects. The biological activities of these biomolecules are extensive, affecting immune cells. These biomolecules demonstrate a wide range of biological activities, including the activation of immune cells, development of immune organs, and enhancement of disease resistance, making them viable substitutes for synthetic immunomodulators (Zhao et al., 2023). TC, a key medicinal plant in Tibetan medicine, is renowned for its wide range of therapeutic benefits, such as antioxidant, anti-inflammatory, antimicrobial, and immune-regulating effects (Lakshmi et al., 2018). Our earlier studies enhanced the bioactive potential of TC by using traditional processing methods in synergy with *Rubia cordifolia* L, leading to the creation of an enhanced polysaccharide (RTCP) that has better immunomodulatory effects than raw TC extracts (He et al., 2023). Despite initial evidence of RTCP’s bioactivity, detailed mechanistic studies are lacking, limiting its translational use and obscuring its complete therapeutic potential.

The spleen functions as a pivotal peripheral lymphoid organ in avian species, playing a central role in antigen presentation. Naïve T lymphocytes within this organ differentiate into T-helper 1 cells (Th1) and T-helper 2 cells (Th2) subsets, governing cell-mediated and antibody-mediated immune functions, respectively. A finely balanced equilibrium between Th1 and Th2 populations is essential for maintaining immune homeostasis, while dysregulation of this balance has been associated with various pathological conditions, including immunosuppression and chronic inflammation (Huang et al., 2011; Talaat et al., 2015). At the core of this regulatory mechanism are the transcription factors T-bet (encoded by TBX21) and GATA-3, which govern the commitment to the Th1 and Th2 lineages, respectively. Consequently, the T-box expressed in T cells (T-bet)/GATA binding protein 3 (GATA-3) expression ratio has become a widely recognized quantitative biomarker for evaluating Th1/Th2 polarization status (Das et al., 2017; Yu et al., 2015).

Our prior network pharmacology analysis identified TC as a modulator of immunosuppression in Chinese yellow quail, suggesting RELA/NF-κB signaling, alongside T-bet and GATA-3, as potential therapeutic targets (Wu et al., 2023). Nevertheless, the precise extent to which RTCP directly modulates T-bet/GATA-3 dynamics remains unexplored. Additionally, although transcriptomic approaches provide unparalleled resolution for elucidating genome-wide regulatory mechanisms, their systematic application to decipher RTCP’s immunomodulatory pathways—particularly in avian models—has yet to be fully realized. This study systematically evaluated RTCP’s capacity to alleviate CTX-induced immunosuppression in Chinese yellow quail, focusing on its effects on spleen organ index, splenic histological structure, serum cytokines and antibody levels, Th1/Th2 balance, and associated transcriptional networks.

## Materials and methods

### Preparation of polysaccharide

TC and *Rubia cordifolia* L. processed *Terminalia chebula* Retz (RTC) were purchased in Chengdu, Sichuan Province, and authenticated by a traditional Chinese medicine expert at Sichuan Agricultural University. Polysaccharides were extracted according to previously established methods by our team (Jingjing et al., 2019; He et al., 2023).

### Experimental design

The present study protocol and animal handling procedures were approved by the Animal Care and Use Committee of Yibin Vocational and Technical College (Approval No. AEC-YVTC-20240318). 120 healthy 21-day-old Chinese yellow quails were randomly allocated into six groups (n = 20). Saline was administered to the blank and model groups, while the treatment groups received RTCP at 0.25, 0.5, 0.75, and 1 g/kg body weight, respectively.

The dosing regimens for CTX injections and oral drug administrations were established based on preliminary experimental data obtained from our laboratory (He et al., 2023). During the initial 72-hour phase, the four RTCP treatment groups received escalating doses ranging from 0.25 to 1 g/kg via oral gavage, while the control groups (blank and model) received equivalent volumes of saline solution. Following three days of pretreatment, intramuscular CTX injections (80 mg/kg body weight) were administered to all groups except the blank group from day 4 to day 7. RTCP administration continued in the treatment groups, and saline was given to the controls until the experiment ended. Grouping and dosages are shown in **Table 1**.

**Table 1:** Treatments of the six experimental groups.

Items	Model	Experimental groups				Blank
CTX (mg/kg) 4~7 d	80	80	80	80	80	0
RTCP (g/kg)1~7d	0	0.25	0.5	0.75	1	0

### Spleen organ index measurement

Upon completion of the seventh experimental day, twenty-four hours post-final gavage administration, five Chinese yellow quails from each group were subjected to systematic necropsy procedures. The animals were first weighed for body mass quantification, then anesthetized using ether induction, and subsequently dissected for splenic tissue extraction. The excised spleens were analyzed gravimetrically using precision instrumentation.

### Serum cytokines and antibody levels

Spleens were aseptically excised from five Chinese yellow quails per experimental group, mechanically homogenized, and subsequently centrifuged to isolate cellular supernatants. Splenic cytokine levels (IL-2, IL-6, TNF-α, and IFN-γ) were quantified using ELISA kits (Wuhan Saipei Biotechnology, Wuhan, China), following the manufacturer’s guidelines.

### Transcriptomics analysis

In this study, splenic specimens were collected in triplicate for each cohort, snap-frozen in liquid nitrogen, and subsequently processed for transcriptomic profiling by a commercial service provider (LC-Bio Technologies). Poly(A)-selected mRNA was fragmented with a magnesium-dependent module (NEBNext^®^) at 94°C for 5–7 minutes. Sequencing was conducted on an Illumina NovaSeq 6000 platform (150 bp paired-end reads). Quality control of raw reads was performed using Cutadapt, and alignment to the reference genome was carried out with HISAT2 (v2.2.1). StringTie and BallGown were used for transcript quantification (FPKM values). DEGs were identified using DESeq2 and edgeR, applying |fold change| ≥2 and FDR <0.05 as criteria. GO and KEGG pathway analyses were utilized for functional enrichment of DEGs.

### Total RNA extraction, and quantitative real-time reverse transcription polymerase chain reaction

RNA extraction protocol: Fresh tissues were stabilized in RNAlater (4°C, 24h) before -80°C storage; RNA integrity was confirmed by Bioanalyzer. Frozen samples were snap-frozen in liquid nitrogen (<5 min post-excision) to prevent degradation. All samples were processed with TRIzol within 1 month, with β-actin RT-qPCR verifying RNA stability.

To detect the relative mRNA expression levels of the genes T-bet and GATA-3 in the spleen and to validate the accuracy of transcriptome sequencing, a total of five Chinese yellow quail per group were sampled for total RNA extraction and subsequent quantitative real-time PCR (qRT-PCR) analysis. The sequences of nine randomly selected genes from the differentially expressed genes obtained through sequencing, along with the T-bet and GATA-3 genes, were retrieved from the online database of the National Center for Biotechnology Information (NCBI) (**Table 2**). Primers for amplifying these sequences were synthesized by Beijing Prime Biotechnology Co., Ltd.

**Table 2:** Nucleotide sequences of PCR primers used to assay gene expression using quantitative real-time reverse transcription PCR.

Gene name	Forward primer	Reverse primer
ACTB	GCGTGACATCAAGGAGAAGC	CACAGGACTCCATACCCAAGAA
C3	TCAGTACCACGTGGCTGTTC	CACCGTGAAGTCCTCGTTCA
HPX	GATGACAGCGGTCGCATCTA	ACATGCGCTTATCCCAGGAG
PIGR	AGTAACCTGGCATGGAACGC	CAGAGCCATCAATCAGGCCA
PSPH	ATGGCTCAAGACGGAGTCCT	TGTCACAGTGCCACCCATAG
PCK1	ATGGACCCTGCATGGGAATC	TTCCCTTGGCTGTCTTTCCG
AMBP	CGTGCTTCGCACCAACTATG	GCTCTGGGCTTCTCCCATAC
GATA-3	ACTACTTGTGTAACGCCTGTGGAC	GTGGTGGTGGTCTGACAGTTAGC
T-BET	CCGACTCACCCAACACC	GTAAGCAGTGACAGCAATGAA

Quantitative PCR analysis was conducted on a CFX96 Touch Real-Time PCR System (Bio-Rad) employing SYBR Green chemistry (SuperReal PreMix Plus, Tiangen Biotech) under standardized conditions: initial denaturation at 95°C (15 min) followed by 40 cycles of 95°C (10 s) and 60°C (30 s). Technical duplicates were implemented for all reactions, with subsequent verification of amplicon sizes through electrophoretic separation. Relative quantification of target transcripts was normalized to avian ACTB (β-actin encoding gene) using the comparative Ct method (the ^2-ΔΔ^Ct method) (Livak and Schmittgen, 2001).

### Statistical analysis

Analysis of all data was initially conducted using Microsoft Excel 2021 and subsequently analyzed using SPSS version 26.0 (IBM Corp., Armonk, NY, USA). In this study, data are expressed as mean ± standard deviation (SD). One-way ANOVA was utilized for the analysis of experimental data, with Duncan’s multiple range test employed for the comparison of means. A P-value of less than 0.05 was considered statistically significant.

## Results

### Spleen organ index

As presented in **Table 3**, dietary supplementation with 0.5 and 0.75 g/kg RTCP markedly enhanced spleen weight and spleen index in Chinese yellow quail compared to the model group (P < 0.05), indicating a positive regulatory effect of RTCP on spleen development. Notably, these values in the 0.5 and 0.75 g/kg RTCP groups were comparable to those in the blank group (P > 0.05), suggesting that RTCP supplementation at these doses could effectively restore spleen parameters to normal levels. In contrast, the model group exhibited a significant reduction in both spleen weight and spleen index relative to all other groups (P < 0.05), further underscoring the potential of RTCP to mitigate spleen-related impairments.

**Table 3:** Effects of RTCP on spleen weight and organ index in immunosuppressed Chinese yellow quail.

Item	RTCP (g/kg)					Blank	P-value
	0(Model)	0.25	0.5	0.75	1.0		
Spleen weight (mg)	37.47 ± 9.23^c^	52.50 ± 7.89^bc^	73.78 ± 17.49^a^	55.66 ± 4.20^b^	48.96 ± 7.06^bc^	83.50 ± 19.42^a^	<0.001
Spleen organ index(mg/g)	0.43 ± 0.10^c^	0.55 ± 0.05^b^	0.83 ± 0.12^a^	0.64 ± 0.03^b^	0.55 ± 0.08^b^	0.84 ± 0.07^a^	<0.001

^a,b,c^Means within a row with no common superscripts differ significantly (*P* < 0.05).

### Histological analysis of splenic structure

**Figure 1:**
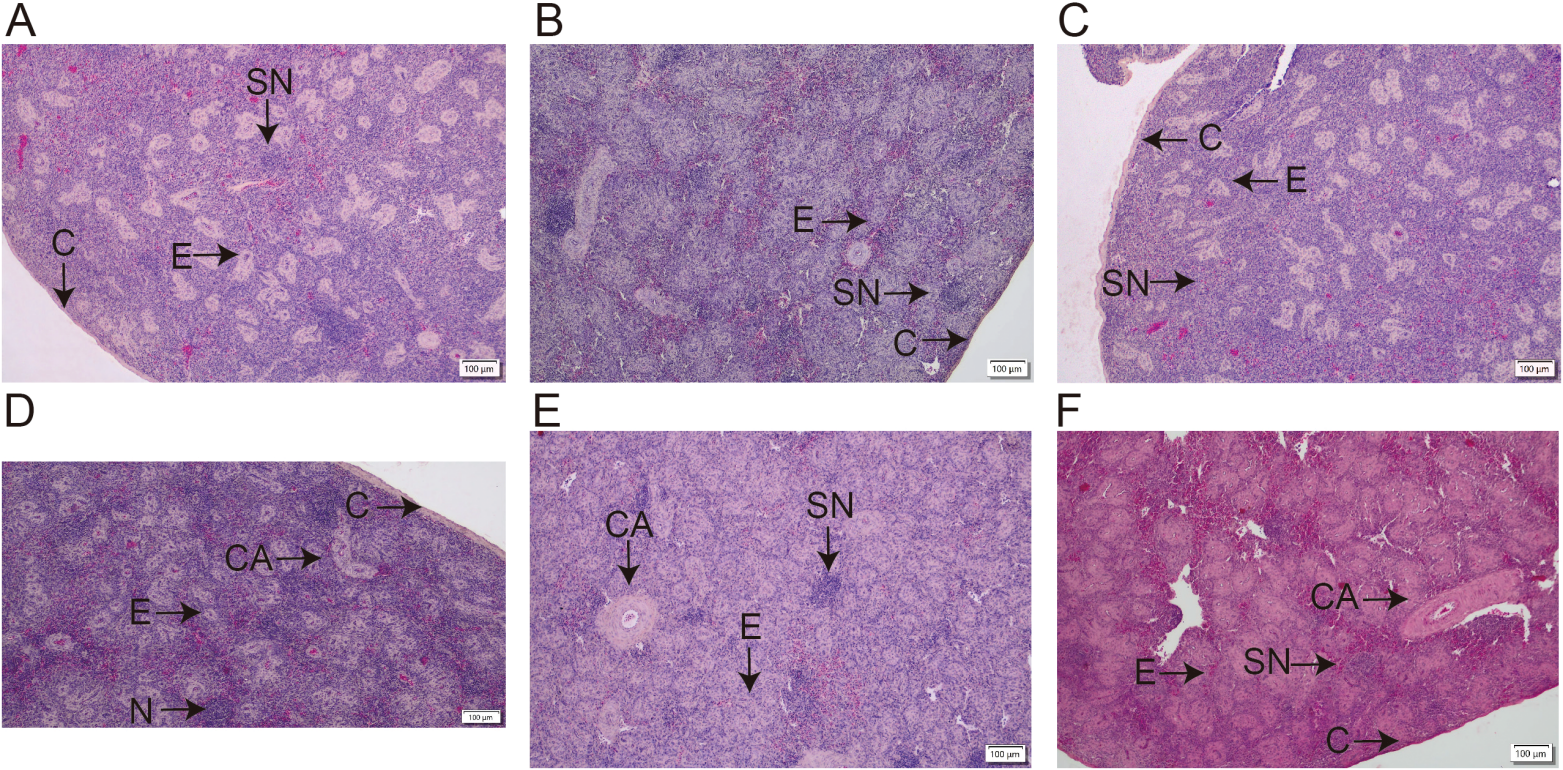
Effects of RTCP on splenic histology in immunosuppressed Chinese yellow quail (hematoxylin-eosin staining, magnification, 100 ×). **(A)** Blank control group; **(B)** Model Group; **(C–F)** 0.25, 0.5, 0.75 and 1 g/kg RTCP processing groups. **(C)** is indicating Capsule; CA represents the central artery (arrow); E represents ellipsoids (arrow); and SN represents the splenic nodule (arrow).

As illustrated in **Figure 1** (100× magnification), distinct morphological differences were observed in the splenic tissue of Chinese yellow quails across experimental groups. In the blank control group (**Figure 1A**), splenic nodules appeared well-developed, with a dense population of erythrocytes within the splenic sinus, suggesting normal physiological function. By contrast, the model group (**Figure 1B**) exhibited markedly atrophic splenic nodules, reflecting compromised splenic structure. Notably, RTCP supplementation at all tested concentrations (**Figures 1C–F**) led to a visible recovery in nodule size compared to the model group, indicating a dose-independent restorative effect.

### Quantitative assessment of splenic nodules

Supporting these observations, **Table 4** provides a quantitative analysis of splenic nodule number and area. Consistent with the histological findings, the model group showed a significant reduction in nodule area relative to the blank control (P < 0.05). Importantly, all RTCP-treated groups (0.25–0.75 g/kg) reversed this trend, with nodule areas significantly larger than those in the model group (P < 0.05). Moreover, the nodule areas in RTCP-fed quails did not differ statistically from the blank control (P > 0.05), implying that RTCP restored splenic morphology to near-normal levels. Interestingly, no dose-dependent differences were detected among RTCP groups, suggesting that even the lowest concentration (0.25 g/kg) may suffice to elicit this protective effect.

**Table 4:** Effects of RTCP on splenic histological structure in immunosuppressed Chinese yellow quail.

Item	RTCP (g/kg)					Blank	P-value
	0 (Model)	0.25	0.5	0.75	1.0		
The number of spleen nodules (number/mm^2^)	7.48 ± 1.02	7.67 ± 1.25	9.00 ± 0.82	9.33 ± 0.47	8.67 ± 0.47	10.00 ± 0.82	0.716
Splenic nodules area (μm^2^)	5123.69 ± 413.17^b^	7987.00 ± 715.84^a^	8994.75 ± 483.52^a^	7409.24 ± 679.68^a^	5513.37 ± 845.65^b^	9122.07 ± 398.08^a^	<0.001

^a,b,^Means within a row with no common superscripts differ significantly (*P* < 0.05).

### GATA-3 and T-bet mRNA expression dynamics

As shown in **Table 5**, CTX-induced immunosuppression profoundly impacted Th1/Th2 transcriptional regulation in Chinese yellow quails, with the model group exhibiting significantly suppressed splenic GATA-3 and T-bet mRNA expression and a reduced T-bet/GATA-3 ratio versus controls (P < 0.05), indicative of Th1/Th2 imbalance. Strikingly, RTCP administration at 0.5–0.75 g/kg counteracted these effects, not only elevating both transcription factor levels (P < 0.05 vs model) but also normalizing the T-bet/GATA-3 ratio to values statistically indistinguishable from healthy controls (P > 0.05). This demonstrates RTCP’s capacity to restore Th1/Th2 homeostasis at effective doses.

**Table 5:** Effects of RTCP on Splenic *GATA-3* and *T-bet* mRNA expression levels in immunosuppressed Chinese yellow quail.

Item	RTCP (g/kg)					Blank	P-value
	0 (Model)	0.25	0.5	0.75	1.0		
GATA-3	1 ± 0^c^	1.19 ± 0.01^b^	1.40 ± 0.08^a^	1.38 ± 0.12^a^	1.21 ± 0.02^b^	1.50 ± 0.25^a^	< 0.001
T-bet	1 ± 0^c^	1.50 ± 0.02^b^	2.02 ± 0.06^a^	2.10 ± 0.04^a^	1.51 ± 0.02^b^	2.25 ± 0.07^a^	< 0.001
T-bet/GATA-3	1 ± 0^b^	1.20 ± 0.16^b^	1.45 ± 0.07^a^	1.42 ± 0.06^a^	1.20 ± 0.11^b^	1.50± 0.08^a^	< 0.001

^a,b,c^Means within a row with no common superscripts differ significantly (*P* < 0.05).GATA-3:GATA-binding protein 3; T-bet: T-box expressed in T cells.

### Immunomodulatory effects of RTCP on humoral and cellular immunity

As detailed in **Table 6**, RTCP administration exerted significant dose-dependent effects on both cytokine and immunoglobulin profiles in immunosuppressed Chinese yellow quails. The model group displayed profound immunosuppression, with serum levels of key cytokines (IL-2, IL-6, IFN-γ, TNF-α) and immunoglobulins (IgA, IgG, IgM) all being significantly depressed compared to the blank control group (P < 0.05), confirming successful establishment of the immunocompromised model.

**Table 6:** Effects of RTCP on serum cytokines and antibody levels in immunosuppressed Chinese yellow quail.

Item	RTCP (g/kg)					Blank	P-value
	0(Model)	0.25	0.5	0.75	1.0		
IL-2	172.9 ± 5.33^d^	195.71 ± 5.00^c^	246.51 ± 6.58^b^	241.54 ± 4.55^b^	208.67 ± 5.49^c^	296.7 ± 6.09^a^	< 0.001
IL-6	12.99 ± 0.76^d^	14.84 ± 1.04^c^	17.86 ± 2.02^b^	17.37 ± 0.92^b^	13.48 ± 1.10^cd^	25.19 ± 1.41^a^	< 0.001
TNFα	41.8 ± 6.58^d^	45.38 ± 5.97^cd^	54.62 ± 5.05^b^	53.5 ± 1.70^b^	50.6 ± 3.64^bc^	71.07 ± 5.22^a^	< 0.001
IFNγ	33.27 ± 1.26^d^	56.18 ± 2.54^c^	77.19 ± 1.97^b^	76.01 ± 2.25^b^	53.70 ± 2.14^c^	80.24 ± 5.28^a^	< 0.001
lgA	181.86 ± 5.45^e^	228.21 ± 6.34^d^	250.36 ± 6.52^c^	262.42 ± 5.40^b^	236.22 ± 5.27^d^	273.93 ± 6.59^a^	< 0.001
lgM	447.06 ± 15.58^e^	482.87 ± 7.95^d^	564.29 ± 18.99^b^	540.88 ± 19.14^bc^	523.69 ± 20.46^c^	642.73 ± 9.54^a^	< 0.001
lgG	1583.83 ± 17.52^f^	1670.54 ± 22.84^e^	1927.5 ± 53.84^b^	1838.28 ± 51.96^c^	1741.1 ± 44.63^d^	2033.73 ± 28.91^a^	< 0.001

^a,b,c,d,e,f^ Means within a row with no common superscripts differ significantly (*P* < 0.05). IL:interleukin; TNFα: tumor necrosis factor alpha; INFγ:interferon gamma.

Notably, RTCP treatment at 0.5 g/kg partially reversed these effects, showing statistically significant elevation in all measured immune markers relative to the model group (P < 0.05). More strikingly, higher doses of 0.75 and 1 g/kg RTCP demonstrated even more pronounced immunorestorative effects, with differences reaching greater statistical significance (P < 0.001 versus model group). This dose-response pattern suggests RTCP may enhance both cellular (via cytokine modulation) and humoral (via antibody production) immune responses in a concentration-dependent manner.

### High-throughput sequencing and data quality control

Comprehensive transcriptome profiling was performed through Illumina NovaSeq™ 6000 sequencing of 18 cDNA libraries representing all six experimental groups (**Table 7**). The sequencing effort generated substantial genomic data, with each sample producing >5 GB of raw sequencing data, cumulatively yielding approximately 700 million paired-end 150 bp reads across all samples. Following rigorous quality control processing that included: Adapter sequence trimming; Low-quality read filtration; Base quality scoring. We retained over 700 million high-quality reads for downstream bioinformatic analysis. The exceptional data quality was evidenced by: Q20 scores exceeding 99.91% (indicating <1% chance of incorrect base calls); Q30 scores surpassing 96.62% (demonstrating <0.1% error probability)

**Table 7:** Quality control statistics of sequencing data.

Sample	Raw Data		Valid Data		Valid Ratio	Q20 (%)	Q30 (%)	GC content (%)
	Read	Base	Read	Base				
B-1	47662940	7.15G	43980534	6.60G	92.27	99.93	96.82	46
B-2	48895258	7.33G	42729594	6.41G	87.39	99.91	97.02	46.50
B-3	45672214	6.85G	29952108	4.49G	65.58	99.93	96.78	46.50
M-1	38976276	5.85G	35738026	5.36G	91.69	99.93	96.86	46.50
M-2	40826074	6.12G	37104284	5.57G	90.88	99.91	96.62	46.50
M-3	45285058	6.79G	40558546	6.08G	89.56	99.93	96.89	46.50
RTCP1-1	46894416	7.03G	42976508	6.45G	91.65	99.92	96.66	47.50
RTCP1-2	46248518	6.94G	41892242	6.28G	90,58	99.92	96.68	46.50
RTCP1-3	48489166	7.27G	41968536	6.30G	86.55	99.92	96.66	47
RTCP2-1	45272422	6.79G	41507416	6.23G	91.68	99.92	97.00	46
RTCP2-2	45789848	6.87G	41400252	6.21G	90.41	99.91	96.94	46.50
RTCP2-3	40350578	6.05G	35103736	5.27G	87.00	99.92	96.85	46
RTCP3-1	45086786	6.76G	41280416	6.19G	91.56	99.91	96.91	46.50
RTCP3-2	47377322	7.11G	41684728	6.25G	87.98	99.91	97.01	46.50
RTCP3-3	50357540	7.55G	46122354	6.92G	91.59	99.93	97.06	46.50
RTCP4-1	40569002	6.09G	36612578	5.49G	90.25	99.92	97.00	45.50
RTCP4-2	39978872	6.00G	36260658	5.44G	90.70	99.91	97.10	45.50
RTCP4-3	44796076	6.72G	40898194	6.13G	91.30	99.91	97.27	45.50

### Integrated analysis of transcriptome sequencing and differential gene expression

**Figure 2:**
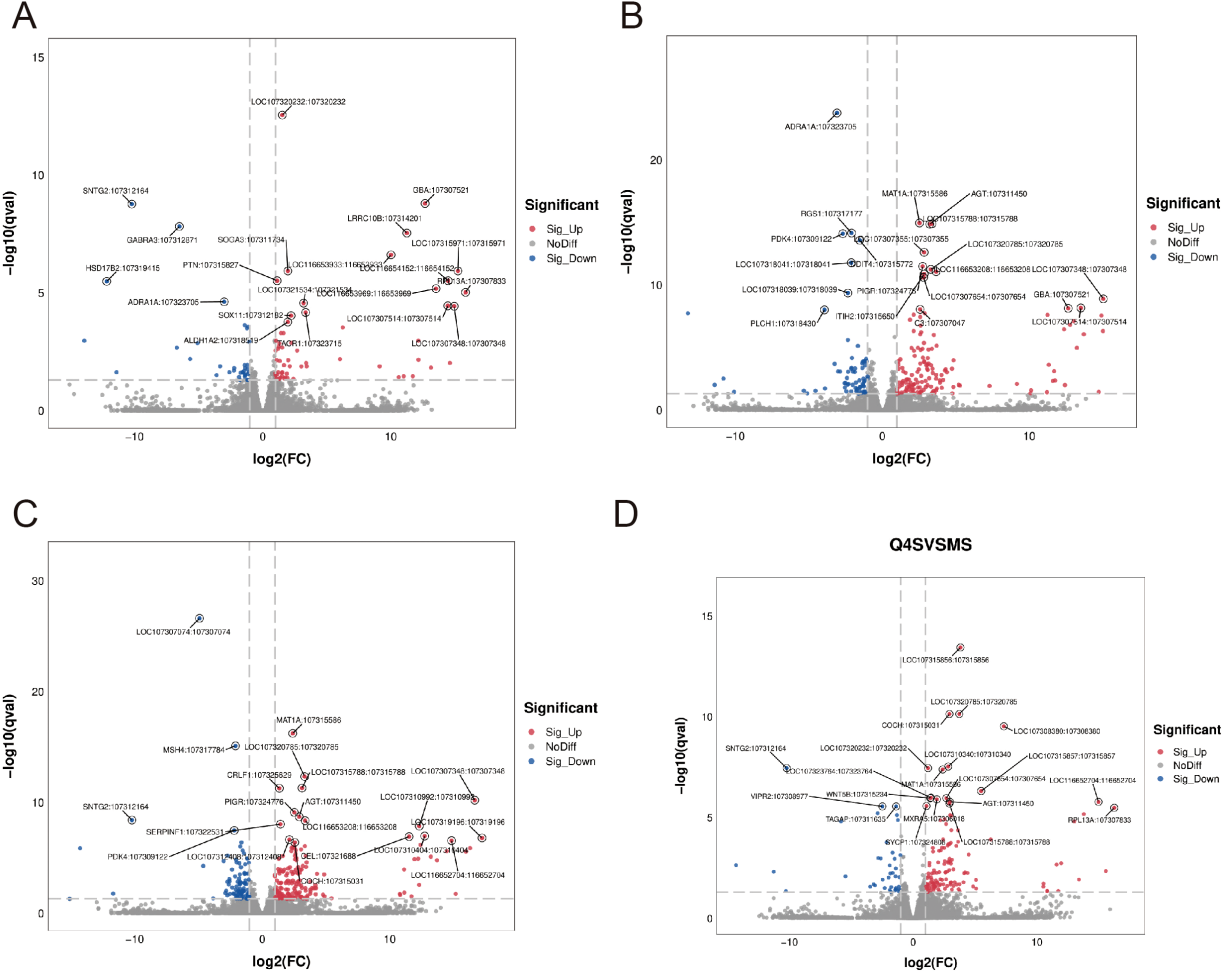
Volcano plots showing differentially expressed genes (DEGs) in response to varying doses of RTCP treatment compared to the model group. **(A)** RTCP 0.25 g/kg group: 94 DEGs were identified, including 59 up-regulated and 35 down-regulated genes. **(B)** RTCP 0.5 g/kg group: 211 DEGs, with 144 up-regulated and 67 down-regulated. **(C)** RTCP 0.75 g/kg group: 274 DEGs, including 175 up-regulated and 99 down-regulated. **(D)** RTCP 1 g/kg group: 167 DEGs, with 129 up-regulated and 38 down-regulated. Red dots represent significantly up-regulated genes, blue dots indicate down-regulated genes, and grey dots denote non-significant changes. DEGs were identified using threshold criteria of |log_2_FC| > 1 and adjusted p-value < 0.05.

Our comprehensive transcriptomic investigation began with high-quality sequencing of 18 cDNA libraries across six experimental groups using the Illumina NovaSeq™ 6000 platform, generating over 700 million high-quality 150-bp paired-end reads (Q20 > 99.91%, Q30 > 96.62%). Subsequent analysis revealed significant dose-dependent transcriptional modulation by RTCP, with 513 total differentially expressed genes (DEGs) identified across treatment groups. The volcano plot analysis (**Figure 2**) demonstrated a clear dose-response relationship: 0.25 g/kg RTCP induced 94 DEGs (59↑/35↓), followed by 211 DEGs (144↑/67↓) at 0.5 g/kg, peaking at 274 DEGs (175↑/99↓) with 0.75 g/kg treatment, before showing a moderated response of 167 DEGs (129↑/38↓) at the highest 1 g/kg dose. This transcriptional pattern, particularly the maximal response at 0.75 g/kg (53% more DEGs than 0.5 g/kg group with a 1.9:1 up/down-regulation ratio), suggests a potentially optimal immunomodulatory dose that balances efficacy with potential regulatory feedback mechanisms. The exceptional sequencing quality (Q30 > 96.62%) ensures the reliability of these dose-dependent genomic findings, which correlate well with our observed phenotypic improvements in immune parameters.

### GO analysis of differentially expressed genes

**Figure 3:**
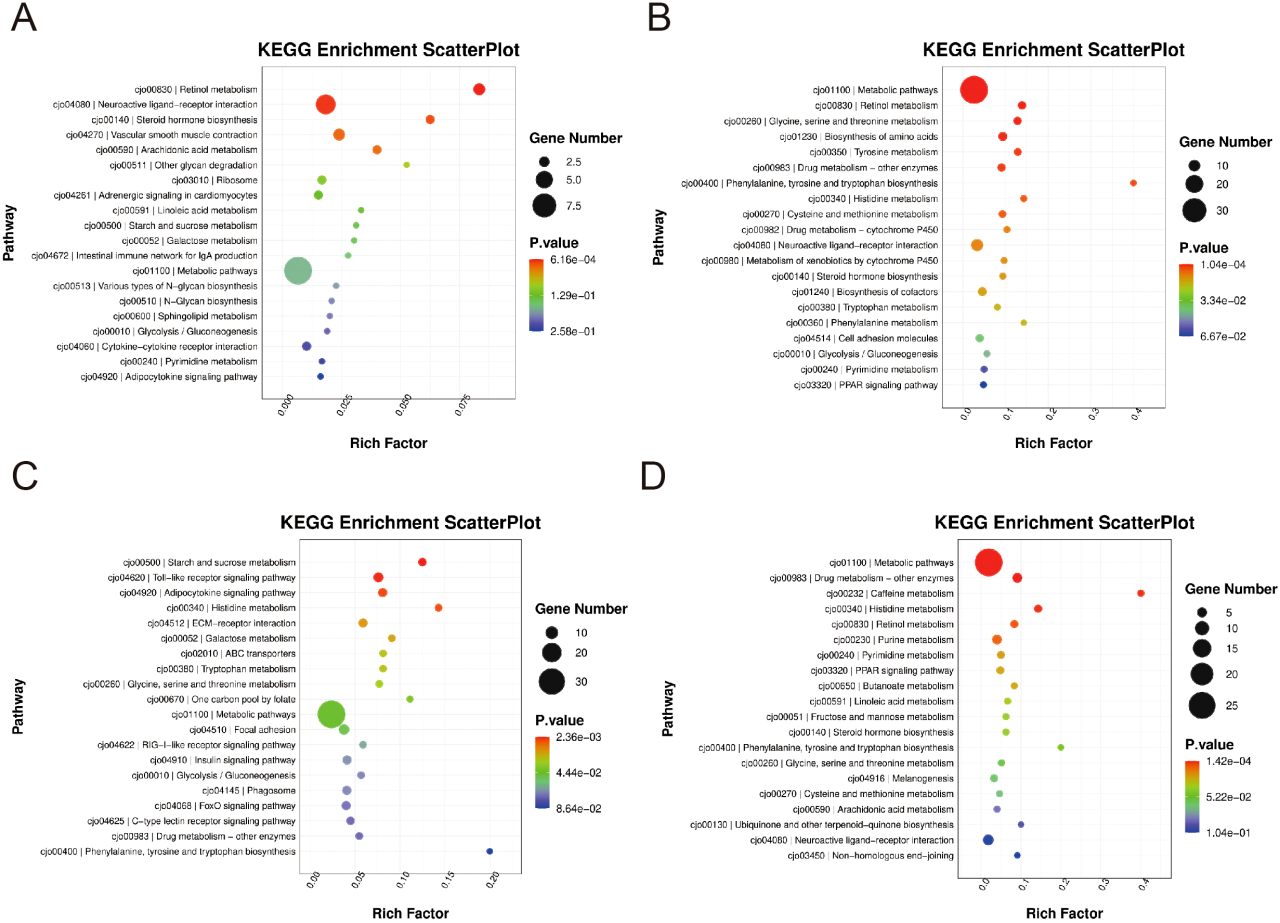
The GO enrichment levels of differentially expressed gene. **(A, B)** In the 0.25–0.5 g/kg RTCP groups, DEGs were mainly enriched in negative regulation of endopeptidase activity, endoderm formation, and coagulation, with functions related to serine-type endopeptidase activity, protease inhibition, and cytokine binding. **(C, D)** In the 0.75–1 g/kg RTCP groups, DEGs were primarily involved in negative regulation of endopeptidase activity and complement activation, enriched in extracellular space and plasma membrane, and associated with protease activity and TNF binding.

The differentially expressed genes in the 0.25-0.5 g/kg RTCP group and the model group of Chinese yellow quail were primarily associated with biological processes such as the negative regulation of endopeptidase activity, endoderm formation, and coagulation. These genes were predominantly enriched in plasma membrane components, blood particles, and extracellular compartments, exhibiting serine-type endopeptidase activity and inhibitor functions. Additionally, they were involved in protease binding, oxygen-related processes, tumor necrosis factor binding, and IL-8 binding (**Figures 3A, B**).

As depicted in **Figures 3C, D**, the differentially expressed genes in the 0.75–1 g/kg RTCP group and the model group of Chinese yellow quail were mainly enriched in biological processes including the negative regulation of endopeptidase activity and complement activation. These genes were functionally localized to the extracellular space, blood microparticles, extracellular domains, and plasma membrane, with molecular functions encompassing serine-type endopeptidase activity, their inhibitors, and TNF binding.

### KEGG analysis of differentially expressed genes

**Figure 4:**
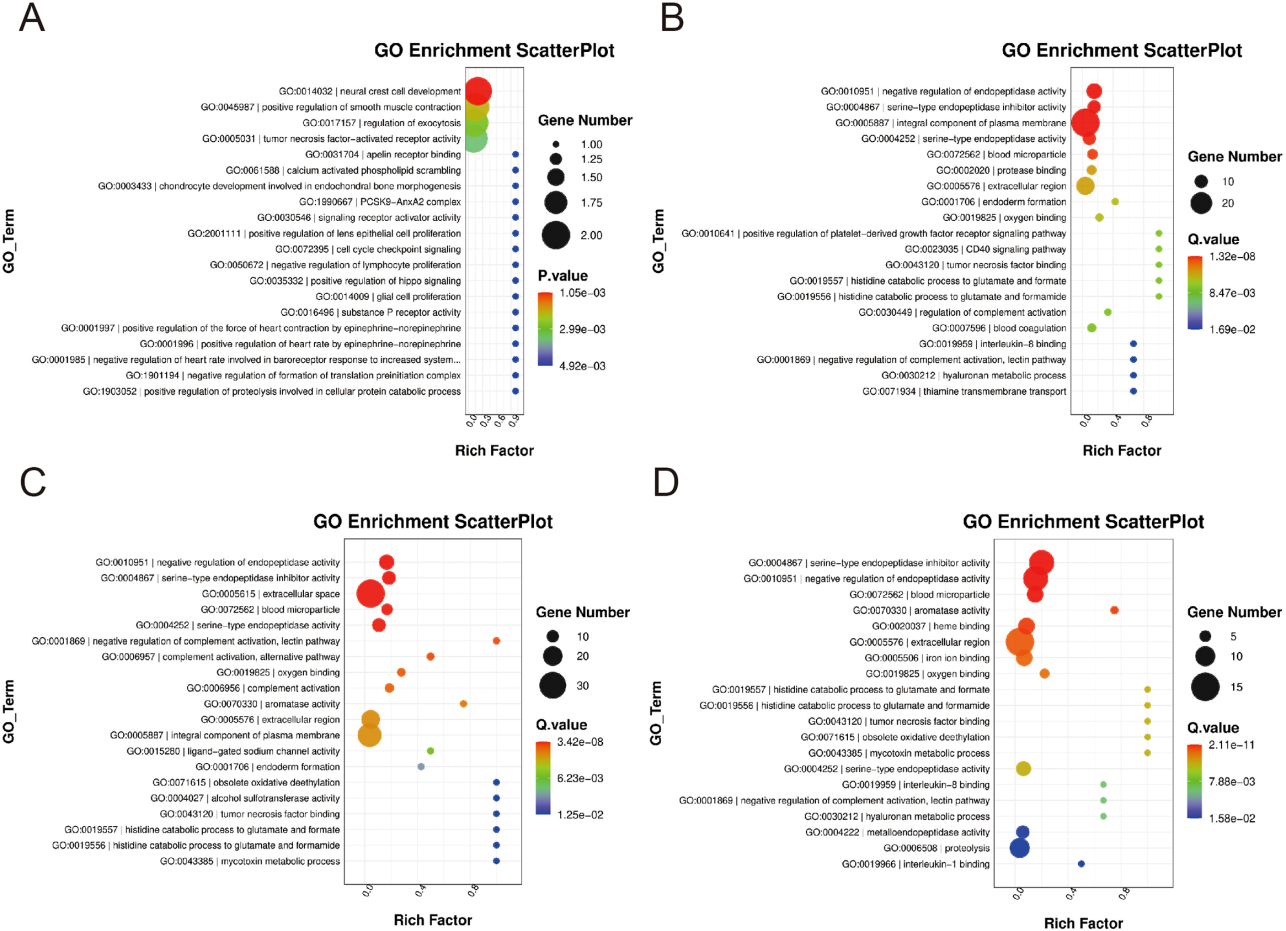
The KEGG enrichment levels of differentially expressed genes. **(A, B)** The differentially expressed genes in the 0.25-0.5 g/kg RTCP group and the model group were mainly enriched in pathways such as glycine, serine and threonine metabolism, retinol metabolism and cofactor biosynthesis. The differentially expressed genes in the **(C, D)** 0.75–1 g/kg RTCP group were mainly enriched in the Toll-like receptor signaling pathway.

To elucidate the biological pathways influenced by RCTP treatment, we conducted KEGG enrichment analysis on differentially expressed genes (DEGs) across the blank group, RCTP dose groups, and the model group. Initial analysis (**Figures 4A, B**) comparing the 0.25–0.5 g/kg RCTP group with the model group revealed that the most significantly enriched pathways were associated with glycine, serine, and threonine metabolism, retinol metabolism, and cofactor biosynthesis, suggesting that lower RCTP doses may primarily modulate metabolic processes. In contrast, when examining the 0.75–1 g/kg RCTP group (**Figures 4C, D**), we observed a distinct shift in pathway enrichment, with DEGs predominantly linked to the Toll-like receptor (TLR) signaling pathway. This finding indicates that higher RCTP doses may exert immunomodulatory effects, potentially through the regulation of innate immune responses. Together, these results demonstrate a dose-dependent influence of RCTP on cellular pathways, transitioning from metabolic regulation at lower doses to immune-related signaling at higher doses.

### Validation of selected differentially expressed genes by qRT-PCR

**Figure 5:**
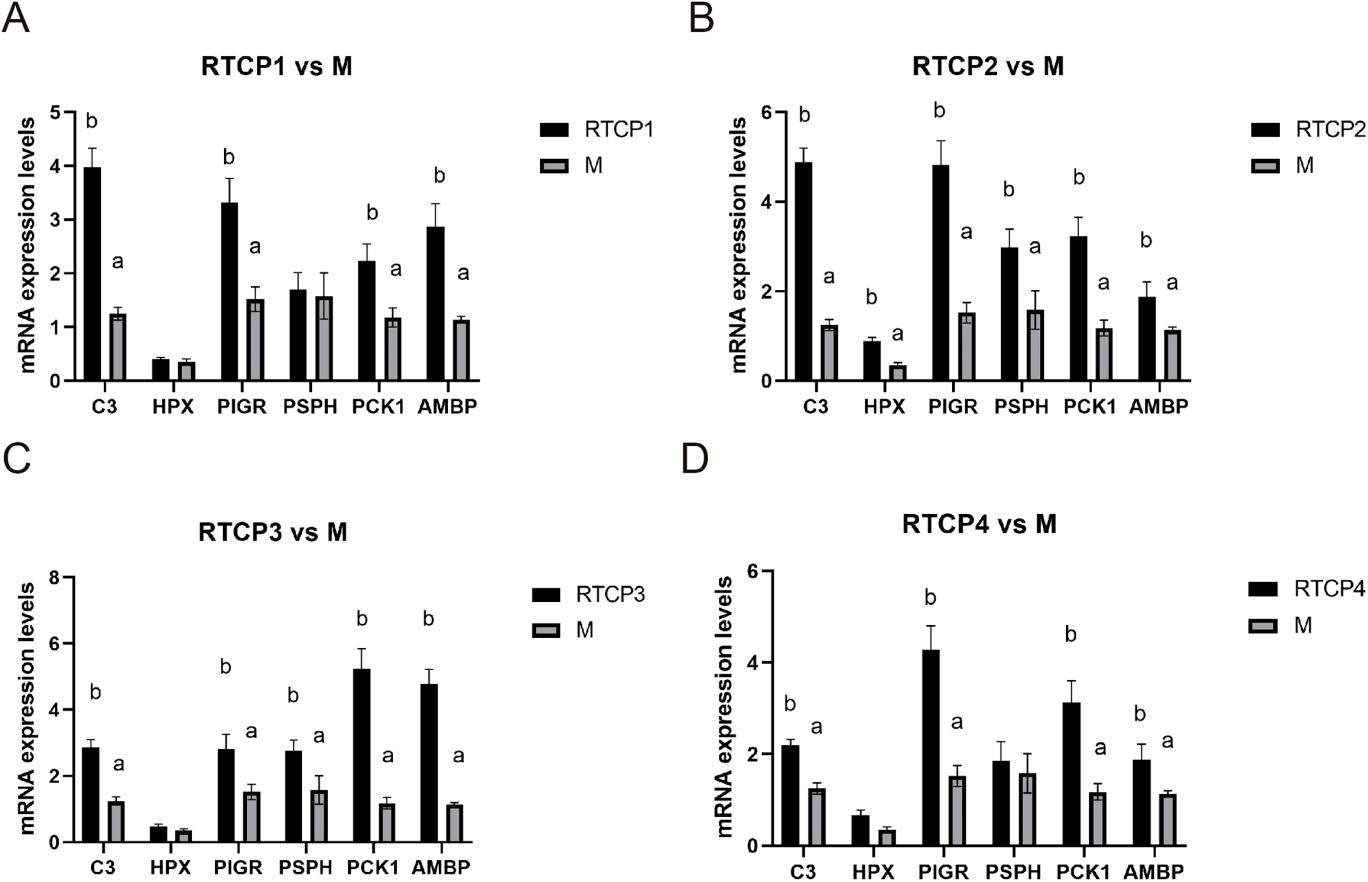
Validation of selected differentially expressed genes by qRT-PCR analysis. **(A)** RTCP1 vs M. **(B)** RTCP2 vs M. **(C)** RTCP3 vs M. **(D)** RTCP4 vs M. The expression levels of C3, HPX, PIGR, PSPH, PCK1, and AMBP in the spleen of Chinese yellow quail were significantly upregulated in the 0.5 g/kg RTCP group compared to the model group (*P* < 0.05), consistent with transcriptome sequencing results.

To confirm the transcriptomic findings, we performed qRT-PCR analysis on six key genes (C3, HPX, PIGR, PSPH, PCK1, and AMBP) in the spleen of Chinese yellow quail. Consistent with the RNA-seq data, all six genes exhibited significantly elevated expression levels (P < 0.05) in the 0.5 g/kg RTCP group compared to the model group (**Figure 5**). Notably, C3, HPX, and PIGR—genes associated with immune and inflammatory responses—showed pronounced upregulation, reinforcing the potential immunomodulatory role of RTCP. Similarly, PSPH, PCK1, and AMBP, which are involved in metabolic processes, also displayed increased expression, aligning with the KEGG pathway analysis that highlighted metabolic regulation at this dose. These qRT-PCR results not only validate the transcriptome sequencing data but also strengthen the biological relevance of the observed gene expression changes, supporting the hypothesis that RTCP modulates both immune and metabolic pathways in a dose-dependent manner.

## Discussion

CTX is an alkylating agent that exerts its effects on DNA. As a result, it has been widely used for modeling immunosuppression. As demonstrated by previous findings, CTX has been demonstrated to alter the homeostasis of the spleen in mice and chickens as follows with decrease in organ indices, disordered structure and abnormal lymphocyte proliferation (Siracusa et al., 2017). In line with these findings, our results report significantly decreased spleen organ indices, splenic nodular area and cytokine contents after treatment with CTX. The outcomes verify the stability of immunosuppression model for the Chinese yellow quail in this experiment and the reliability of CTX induced model for mimic the cross-species immunosuppression.

It has also been well recognized that plant-based polysaccharides possess the effects of regulation of immunity. For instance, administration of 0.5 g/kg/day RTCP can obviously ameliorate the CTX-induced atrophy of spleen of Chinese yellow quail by increasing the spleen weight (reaching 89.2% of the control), organ index and the area of splenic nodules. The increase in spleen indices and area of spleen nodules suggests improvement of immunity both at humoral and cellular level which is consistent with the previous report (Zuidhof et al., 2014). The findings above are similar to those on the protective effects of phytopolysaccharides on the immunological organs of other chicken model (Cheng et al., 2024) indicating that 0.5 g/kg/day RTCP significantly counteracts the CTX-induced suppressing effect on immune organ function and induces the reversion of the morphology of spleen.

Serum production of IgG, IgM, IgA by B lymphocytes in response to antigen represents a measure of the humoral arm of the immune system, while cytokine profiles mainly produced by T cell constitute a marker of cellular immune response. The RTCP administration showed that CTX can re-establish both serum immunoglobulins (IgA, IgG, IgM) and Th1 type cytokines (IL-2, IL-6, TNF-α, IFN-γ), i.e., RTCP shows also the capacity to recover humoral and cellular immunity. This corresponds to known effects on T/B-cell proliferation and secretion observed by plant polysaccharides in birds to recover CTX -induced lymphocyte dysfunction of geese (Li et al., 2018); chickens (Fan et al., 2013); and chick (Cheng et al., 2024).

T-bet (TBX21) and GATA-3 are known to be fundamental players in T1/Th2 polarization by virtue of their expressions in cellular versus humoral immunity pathways (Kidd, 2003). Here, T-bet and GATA-3 showed synchronized up-regulation with those of cytokines and immunoglobulins in RTCP-treated quail which suggests possible co-stimulation between Th1/Th2. Detailed analysis of cellular proliferation in this case is to be seen in forthcoming studies. Targeting complement-T cell interactions is a double-edged sword, balancing efficacy and toxicity. Preclinical studies show promise (C5aR1 blockade suppressing Th17 cells, CD46 agonism boosting antitumor immunity), but clinical translation faces challenges: (1) infection risks, (2) cytokine toxicity from overactivated T cells, and (3) immunosuppression in chronic diseases. Strategies to improve safety include tissue-targeted delivery (CR2-fusions), combining checkpoint inhibitors (to reduce exhaustion), and biomarker-guided dosing (monitoring C3a/C5a or T cell exhaustion). The FDA-approved C5aR antagonist avacopan proves this balance is possible—effective in vasculitis without broad immunosuppression—though patient stratification remains key to minimizing off-target effects. Mechanistically, treatment with RTCP also recovered the balance of transcription for T-bet and GATA-3, which are main regulators for Th1/Th2 differentiation. The ratio (normalized with respect to internal standard and sample RIN values) of T-bet to GATA-3 reflects the recovery of balance between Th1)and Th2 responses, an important determinant of immune homeostasis (Ying et al., 2020). These observations are in agreement with earlier studies from Atractylodes macrocephala Koidz (PAMK) (Guo et al., 2012) and Tremella polysaccharide (Zhou et al., 2018) in immunosuppressed mouse models.

Transcriptomic profiling additionally revealed that treatment with RTCP increased complement (C3, C5), HPX and immunity related genes (PIGR, PSPH), whereupon C3 is a β-globulin synthesized by, for instance, macrophages, monocytes and lymphoid tissues. As an integral part of innate immunity, C3 plays a role in activation of all the complement pathways, allowing for antigen presentation and T-cell activation via binding of fragments to associated loci on the surface of the T cell (Sahu and Lambris, 2001; Yuan et al., 2011). C5, upon cleavage by C5-converting enzyme into C5a, enhances the body’s defense mechanisms, promotes T-cell proliferation induced by antigens and alloantigens, and stimulates antibody production by B-cells (Guo and Ward, 2005). Moreover, HPX, an acute-phase response protein, is activated via the JNK/SAPK pathway, leading to inhibition of apoptosis, promotion of Th1 activation, and regulation of the Th1/Th2 balance. The complement system regulates T cell responses through direct and indirect mechanisms. Anaphylatoxins C3a/C5a drive Th1/Th17 differentiation and suppress Tregs via mTOR-mediated metabolic reprogramming. CD46 costimulates T cells through mTORC1 signaling. Indirectly, C3b opsonization enhances APC-mediated T cell activation, while C5a-C5aR1 in tumors promotes CD8+ T cell exhaustion. Together, these findings highlight complement as a pivotal modulator of T cell immunity in health and disease (Arredouani et al., 2003).

The aforementioned transcriptional changes were accompanied by the upregulation of complement components (C3, C5) and HPX. These alterations collectively enhance the proliferation and activation of T lymphocytes and stimulate antibody production by B lymphocytes. Such molecular insights align with the observed histological and functional recovery of the spleen, indicating that RTCP potentiates both innate and adaptive immune pathways to counteract CTX-induced immunosuppression.

Nevertheless, certain limitations must be acknowledged. The restricted dose range of RTCP (0.25–1 g/kg) precludes definitive conclusions regarding optimal efficacy, thereby necessitating dose-response studies over extended durations. Additionally, while splenic transcriptomics provided valuable mechanistic insights, complementary analyses of gut-associated lymphoid tissue or systemic metabolite profiling could further elucidate broader immunometabolic interactions. Future research should also investigate RTCP’s bioavailability and its interaction with gut microbiota, a critical factor influencing polysaccharide bioactivity as reported by Stanley and Sundaram (2014).

**Figure 6:**
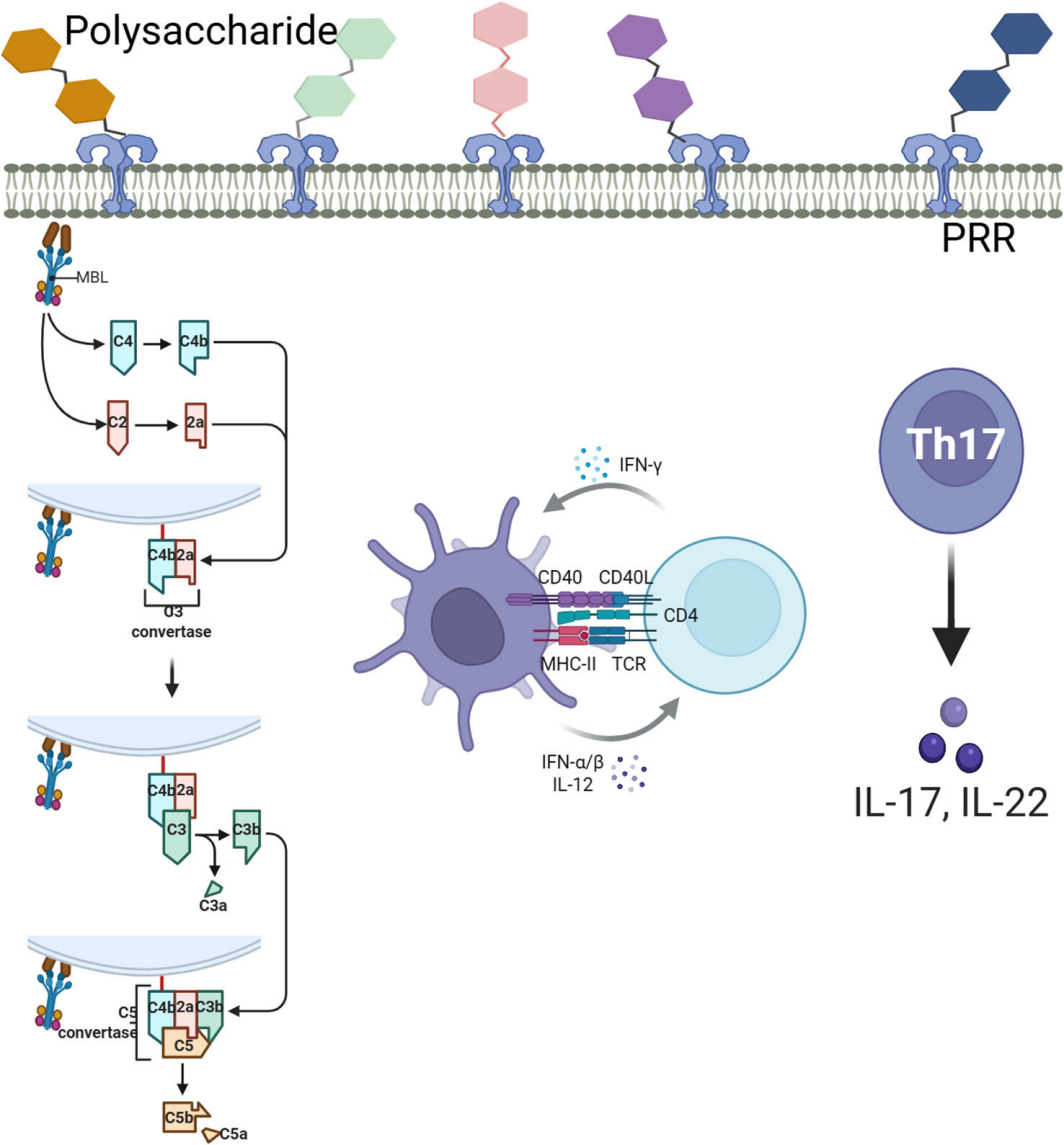
Schematic diagram of polysaccharide recognition, complement activation and T cell differentiation.

Structural analysis showed RTCP contains β-glucan and mannose motifs, typical of fungal/bacterial polysaccharides. Transcriptomics revealed three key PRRs: TLR4 (binds polysaccharides via MD2), Dectin-1 (β-glucan receptor triggering Syk/CARD9), and MBL2 (activates lectin complement via MASP). These PRRs activate NF-κB, matching the observed cytokine increase (IL-6, TNF-α). Dectin-1 and TLR4 synergistically boosted macrophage C3 production, likely driving complement activation. Bioinformatics suggests RTCP-PRR interactions, but direct binding studies are needed for confirmation (**Figure 6**).In summary, RTCP protects immune organs by enhancing splenic indices and the area of splenic nodules, thereby promoting both cellular and humoral immunity while maintaining the balance between Th1 and Th2 responses, which alleviates CTX-induced immunosuppression. Transcriptomic analysis revealed that RTCP upregulates the expression of C3, C5, and HPX, which facilitates T lymphocyte proliferation and activation as well as stimulates B lymphocyte antibody production. These findings indicate that RTCP serves as a promising immunomodulatory agent capable of effectively counteracting immunosuppression through the coordinated regulation of dual immune pathways.

## Data Availability

The original contributions presented in the study are included in the article/supplementary material. Further inquiries can be directed to the corresponding author.
